# Climate change, human health, and the exposome: Utilizing OMIC technologies to navigate an era of uncertainty

**DOI:** 10.3389/fpubh.2022.973000

**Published:** 2022-09-21

**Authors:** Hana Abdelzaher, Sherouk M. Tawfik, Ahmed Nour, Sarah Abdelkader, Shaimaa Tarek Elbalkiny, Mohamed Abdelkader, Walaa A. Abbas, Anwar Abdelnaser

**Affiliations:** Institute of Global Health and Human Ecology, The American University in Cairo, New Cairo, Egypt

**Keywords:** climate change, exposome, omics, health, transcriptomics

## Abstract

Climate change is an anthropogenic phenomenon that is alarming scientists and non-scientists alike. The emission of greenhouse gases is causing the temperature of the earth to rise and this increase is accompanied by a multitude of climate change-induced environmental exposures with potential health impacts. Tracking human exposure has been a major research interest of scientists worldwide. This has led to the development of exposome studies that examine internal and external individual exposures over their lifetime and correlate them to health. The monitoring of health has also benefited from significant technological advances in the field of “omics” technologies that analyze physiological changes on the nucleic acid, protein, and metabolism levels, among others. In this review, we discuss various climate change-induced environmental exposures and their potential health implications. We also highlight the potential integration of the technological advancements in the fields of exposome tracking, climate monitoring, and omics technologies shedding light on important questions that need to be answered.

## Introduction

Anyone alive during the twenty-first century cannot deny that the Earth is suffering. The planet's health is rapidly deteriorating as evidenced by the extinction of entire species, the acidification of our oceans, scarce water resources, degradation of land, and unpredictable weather changes (1). These changes are derived from human abuse and unsustainable exploitation of our planet's resources. These practices may have lasted us for centuries as showcased by the rise in human health and wellbeing along the ages, yet there comes a tipping point. With this deterioration of our planet's health in mind and with the anticipated detriment of said deterioration on human health, a novel concept appeared. A holistic approach toward viewing human health and the health of our planet as one started taking shape and was termed “planetary health” (2). This approach highlights that the dangers lying ahead for the human population are not merely derived from a single factor, yet from all changes resulting from anthropogenic activity. In order to tackle these issues, planetary health recommends the orchestration between human, political & economic systems and their natural counterparts (3).

One aspect that is greatly endangering planetary health is climate change. Climate change is defined as the long-term alteration in the earth's climate (4). It has taken nearly a century of research and gathered data to convince the scientific community that human activity will change the climate of the planet (5). In the eighteenth century, experiments suggested that Carbon Dioxide (CO_2_) and other gases can accumulate in the atmosphere and insulate the earth (6). CO_2_ readings provided some of the first data to support the Global Warming theory by the late 1950's (7). The Intergovernmental Panel on Climate Change (IPCC) was established in 1988 after the first World Climate Conference (WCC) was held and both the conference and panel remain to be major milestones and references in the battle against climate change (8, 9).

The second WCC witnessed the appeal for the creation of a global treaty on climate change leading to the start of the UN General Assembly discussion of a framework convention (10). This led to the first conference of the parties (COP1), a longstanding tradition with COP27 scheduled to take place in Sharm ElSheikh, Egypt in 2022 (10). Climate modeling has recently revealed that Global Warming is real and that it has a slew of disastrous consequences (11). The repercussions of 1.5 and 2°C global warming have been highlighted since the Paris Agreement, yet the rate of warming has regional effects as well (12, 13). With the advent of sequencing technologies, a significant paradigm shift was introduced into how we perceive gene expression and protein function. Biochemical pathways implicated in the pathogenesis of chronic illnesses were identified. However, it quickly became evident that the epidemiology of most of these diseases consisted of both genetic variants and environmental risk factors/exposures (14, 15). Bearing this assumption in mind, researchers identified the need for precise monitoring methods to outline an individual's lifetime history of environmental exposure and correlate it with their genetic makeup and changes therein, if any.

With the impending changes anticipated to occur in our environment due to the anthropogenic shifts of climate change, an uncertain and highly volatile future of human health and exposure is beginning to take shape. To improve our understanding of climate stabilization and its consequences on our health, we need a new framework of experiments. Therefore, reliable evaluation of direct and indirect climate change effects and their consequences on human health is required for the total exposure assessment (16). This improved understanding is critical to guide the policies and systems that will aid us in diverting the course of the impending negative health impacts of the ongoing climate crisis (16).

## The exposome: A novel approach to environmental exposure

The Exposome is a newly evolving field that can assess total exposures such as chemical and biological agents, and external environmental stimuli throughout populations' lifetime (17). The exposome is provides a complete history of individuals' lifetimes of environmental (non-genetic) exposures that include pollution, toxicants, diet, lifestyle choices, and even socioeconomic status (17). The exposome is defined to include two main domains: internal and external environmental exposures as indicated in Table 1 (18). The internal environmental exposure is the domain of internalized exogenous exposures, which includes the fraction of biologically active chemicals as non-nutrient environmental molecules and the internal endogenous nutrient exposures such as gut microbiota, gut microflora, and their accompanied metabolites, inflammatory elements, and oxidative stress (19). While the external exposome exposure is divided into general and specific. The specific external environment is expressing the domain addressing specific contaminants, diet, physical activity, tobacco, infections, or medication (19). On the other hand, the general external environment is the domain of external environmental factors such as the urban environment, climate factors, pollution, social capital, and stress (20).

**Table 1:** Types of exposomes external and internal exposures.

**External exposures**		**Internal exposures**	
**General external exposures**	**Specific external exposures**	**Internalized exogenous exposures**	**Internalized endogenous exposures**
Urban environment Climate factors Air pollution Social stressors	Chemical Contaminants Diet exposures Medication	Fractions of non-nutrient molecules	Gut microbiota Accompanied metabolites Oxidative stress

## Climate change-induced environmental exposures on health

From a planetary health perspective, models have shown that climate change will strongly impact human health and quality of life. Negative outcomes on food security due to decreased crop yields will cause undernutrition in severely affected areas. Water scarcity and decreased fresh water equity, altered zoonotic disease patterns due to shifting weather patterns and toxic chemical exposure due to pollution were also found to be a direct result of anthropogenic shifts. The most obvious result of climate change; extreme weather events, are anticipated to lead to the displacement of entire populations which comes with its own set of health effects and accompanying mental health impacts.

The effects of climate change on humans could be caused directly as well as indirectly. For example, climate change is expected to lead to significant changes in the growth, development, and activity of organisms, including seasonal pests (e.g., insects, mites, mice, fungi, bacteria, and nematodes) and unwanted plants (21, 22). On one hand, this will impact human health directly through the alteration of nutrition as well as disease patterns (22). Scientists believe that climate change and the attendant global warming effects will encompass variations in atmospheric carbon dioxide (CO_2_) and temperature as well as changes in precipitation, soil quality, productivity, nitrogen deposition, and plant diversity (23, 24). Interestingly, temperature increases and precipitation changes are the main climate change-induced effects determining pest growth, abundance, and infectivity (25, 26). In other words, increasing temperatures are known to enhance insect spread, migration, reproduction, and growth rates.

It is believed that rising temperatures lead to the geographical expansion of many species of weeds (27). Higher CO_2_ concentration is also believed to increase weed severity and herbicide resistance due to the CO_2_-enhanced fertilizing effect and water use of weeds compared to other crops (28, 29). Such tolerance to herbicides occurs as a result of increased leaf thickness and partially closed surface stomata, thus reducing herbicide uptake and efficacy leading to the expansion in their use (30).

This will also affect human health indirectly as the use of pesticides, herbicides, and other chemicals is expected to rise. With these changes, humans will easily be exposed either occupationally by direct skin contact and inhalation, or consumingly by ingestion of contaminated crops. Moreover, extreme climatic conditions in terms of humidity and precipitation promote the proliferation of bacteria and plant pathogens as well as germination, reproduction, and activity of spores, resulting in high disease risk and severity (31, 32). It is well-known that plant diseases are essentially influenced by climatic conditions such as humidity, rainfall, temperature, and radiation (33). In addition, the increase in temperature will affect the productivity of the plant, and thus lead to the expansion of the use of pesticides to compensate for plant losses (34, 35).

There are many health concerns that related to several environmental exposures. For instance, it is estimated that polycyclic aromatic hydrocarbons (PAHs) have increased as a result of environmental exposures (36). PAHs are molecules with 2–7 benzene rings incorporated in their chemical structure, and are released during the combustion of carbon-containing materials (such as biomass materials, coke, and petroleum fuels) (36).

High exposure to PAHs has been associated with a higher risk of ischemic heart disease and cancer among workers with high exposure (e.g., foundry and asphalt kiln workers) (37). It has also been found that the negative health effects of PAHs affect those who work near combustion sources (e.g., prairie firefighters, traffic police, garage workers, and taxi drivers) (37). Several studies discussed the potential changes in PAHs concentrations associated with climatic changes (38). In a Korea-limited study, predicted an increase in PAHs concentrations in the atmosphere and water with future climate change. As most occupation-focused exposure studies rely on current reported emissions of PAHs to predict the future, exposure to PAHs from climate change is estimated to vary according to local sources (39). For example, exposure of outdoor workers (particularly drivers, traffic police, and garage workers) to PAHs will likely be decreased due to expansion of the use of cleaner-burning fuels and electric vehicles in transportation sectors. However, exposure of prairie firefighters to PAHs is expected to increase due to the expected increase in wildfires. Environmental chemicals of variant sources are expected to rise dramatically as a consequence to climate changes and associated natural catastrophes such as floods and wildfires. Exposure to chemical spills from floods and wildfires, soil dust following agricultural tilling and harvesting, and storm-induced chemicals is estimated to increase in the future (40). Soil dust has been associated with a variety of health problems; however, it is expected to increase in hot and dry climatic conditions and enhance exposure to particulates and chemicals (41, 42).

Veterinary professionals, farmers, and animal food producers will be increasingly exposed to veterinary medicines, used to mitigate the global warming-induced outbreaks in livestock (43). In addition, climate changes are likely to affect the transport pathways (in the form of storm runoffs and precipitation increases) of chemicals and particulates, and that subsequently will vary according to the chemical characteristics of the particulate matter (solubility, volatility, and hydrophobicity) and the form of dispersion (particulate, particle-bound, and dissolved) (44, 45). Furthermore, temperature increases above thermoneutral temperature, as well, can aggravate the associated health impacts of most contaminants *via* disrupting thermoneutrality and raising the ambient temperature (46). Sweating surges associated with high temperatures can speed up the clearance rate of many toxicants (47). Interestingly, previous studies have shown that the acute toxicity of many chemicals is exacerbated by heat stress (48).

## Health effects of climate change-induced environmental exposures

**Figure 1:**
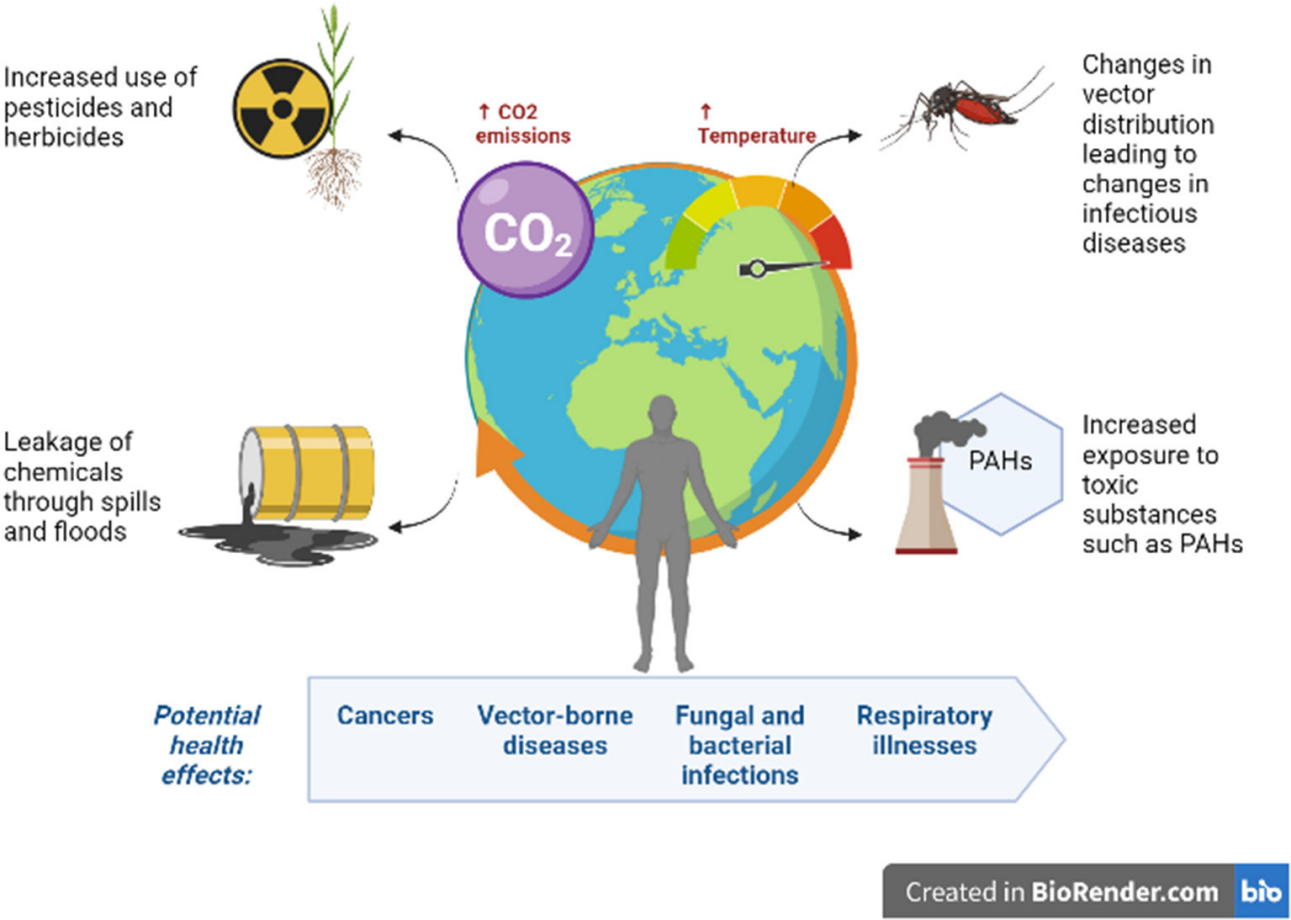
Climate change-induced environmental exposures and their potential health effects.

The exposome is a summation of the biological exposures (external exposome) and biological effects (internal exposome) which can affect macromolecules such as structural changes (ex: mutations and epigenetic modifications) and protein dysfunctions as shown in Figure 1 (49). Biomarkers of exposure studied include markers of exposures, markers of effect, and markers of susceptibility (49). Allergies development and exacerbations are largely influenced by external exposures (50). It has been found that the recent increase in the occurrence of allergic diseases is caused mainly by environmental changes (51).

Allergic diseases specially skin and respiratory allergies are conditions that can be attributed to the changes in particulate matter in the due to pollution and changes in biodiversity leading to change in the external exposome and subsequently internal exposome (50). One of the external exposures heavily studied is aero allergens which are divided into two categories: specific exposures (such as physical, chemical, and biological exposures) and non-specific external exposures (climate, biodiversity, and socioeconomic status) (50, 52, 53). Climate is a non-specific external exposure which directly affect allergies or indirectly affects the risk factors (54). For example, heat waves were found to be positively associated with incidence of hay fever in the United States (55, 56).

Climate change, urbanization and lack of biodiversity affects the nature and concentration of air pollutants subsequently increasing allergic diseases and numbers of allergic patients worldwide (50). Examples of specific exposures are air pollutants such as nitrogen dioxide (NO_2_), Ozone (O_3_), volatile organic compounds, and particulate matter (PM) with different sizes and composition. PM from diesel exhaust contains polycyclic aromatic hydrocarbons (PAHs) (50, 57). Evidence shows that exposure to fine PM, NO_2_, and O_3_ causes exacerbations of asthma and atopic dermatitis in addition to the new incidence of asthma (58, 59).

Climate change due to deforestation increased human activity, desertification, wildfires, and traffic leads to the increase of all of these pollutants both indoors and outdoors affecting human health (60). Additionally, increased temperature due to climate change increases the rate of fungi growth (61). Moreover, extreme weather conditions can cause the breakdown of the buildings allowing more water leaks into the walls increasing dampness and mold growth (41, 62). Studies have found that exposure to indoor dampness and molds especially during infancy can be associated with exacerbations of allergic diseases including persistent asthma and allergic rhinitis which was confirmed by several studies (63, 64).

On the other hand, skin health and aging can be influenced by pollution and climate change. Both internal exposome and external exposome, including our lifestyle, external factors such as pollution, and our skin microbiome can influence our skin health and skin aging (65). Dry conditions lead to increased epidermal permeability and increased secretion of inflammatory mediators. Also, exposure to dry cold temperature increase skin irritation and make it more prone to wrinkles (65, 66). It is hypothesized that climate change can cause an increased risk of vector-borne diseases (67). The diseases of concern are malaria, dengue fever, zika, chikungunya, and yellow fever, all of which are mosquito-borne diseases in tropical and subtropical climates which makes their transmission affected by climate changes (68). In addition to the pathogen transmission being directly affected, socioeconomic status affected by climate change can play a role too indirectly (67). Climate change leads to increased temperature, change in the rate and nature of extreme weather events, changes in humidity and total rainfall (69). All these factors can affect the survival, maturation, and life cycle of the vectors. These can result in changes in the seasons and geographical range of these vector-borne diseases and their risk level (69, 70). Moreover, climate change can indirectly affect the risk level by changing the socioeconomic status which reflects the risk of people being bitten more by vectors and the rate of diagnosis and treatment if bitten (70, 71).

One piece of evidence on the effect of climate change on vector-borne diseases, however not of a public health significance, is the emergence of the Blue-tongue virus (BTV) in Europe coming from North Africa due to increased temperature in Europe (70, 72). A literature review conducted by Rickerts et al. stated that climate changes can also cause changes in the epidemiology of human systemic fungal infections such as endemic mycoses and cryptococcosis (73). An increase in incidence rates had been reported in some areas and the underlying mechanism can be attributed to changes in temperature, precipitation, extreme weather events among other factors (73). All these lead to changes in the environment of these pathogenic parasites (74).

Global warming and increased temperatures especially in urban areas is said to affect metabolic hormones (75, 76). A cross-over trial on non-obese healthy adults in Cyprus concluded that living in an area of a lower temperature for a short period of time significantly reduced leptin levels, a neuroendocrine hormone (77). Though, these results need further confirmation. Another cohort study has found a linear positive correlation between long term environmental exposure to Polychlorinated biphenyls (PCB 153), an air pollutant with an estrogenic action, and increased risk of estrogen receptor positive breast cancer in women (78).

With the vast wealth of data currently available, a centralized approach to exposure tracking is quickly becoming a priority. The exposome reveals the hidden elements behind the role of external environmental factors in human diseases and due to the dynamic nature of environmental exposures and their effects, measuring the exposome and its outcomes is considered a challenge in epidemiological studies (79). This requires newer technologies such as internal biomarkers associated with the exposure, omics, sensors, and geographic or spatial information, which allow for a more comprehensive understanding of the exposome (19).

## OMICs technologies and study designs for the monitoring of climate change-induced environmental exposures

Exposure research received less attention than it deserved in various areas throughout the era of the human genome project. Understanding the fundamental causes of disease pathology and accelerating precision health were two promises made by the human genome project (80). While the study of genome has substantially increased our understanding of the causes of many diseases, it has also revealed that genetics accounts for a much smaller percentage of disease susceptibility than previously thought (81). Environmental exposures, as well as their interactions with an individual's genetics and physiology, appear to make up the remaining components (82).

As a result, environmental health research utilizing novel comprehensive approaches is crucial for identifying modifiable exposures and understanding the associated disease pathways. This type of work has the potential to speed up environmental health studies and help the general population accomplish broader health goals, particularly for vulnerable individuals who may not benefit directly from precision health (83). There are several tools and technologies that are used in the exposomes assessment such as environmental sensor technologies, geographic information system-based environmental tools such as air pollutants, biomarkers, and omics technologies, which provide a window of gathering several individual exposures in one measurement (19, 84) (Table 2).

**Table 2:** Omic technologies and study designs for the monitoring of climate change induced environmental exposures.

**OMICs technologies**		**References**
Metabolomics	Allow investigation of metabolites in the quantitatively dominant species Entails the simultaneous and quantitative detection of large numbers of small molecules in biological systems	(92, 93, 108, 109)
Proteomics	Revealing proteins in the context of more quantitatively dominant species. Proteomics can be used to analyze the toxicity of pollutants and byproducts in order to early detect potential environmental risk factors	(92, 114, 116)
Transcriptomics	Focuses on gene expression at the RNA level and provides genome-wide data on gene structure and function to uncover the molecular mechanisms underlying certain biological processes. Entire set of RNA transcripts in a certain cell type or tissue at a specific developmental stage.	(124–127)
**Exposomes initiatives**		
EXPOsOMICS	European initiative for water and air pollution. It relys on finding relationships between external environmental exposures and biochemical and molecular alterations	(130–132)
HEALS	European project aims to assess the internal exposure of individuals	(133)
HERCULES	Launched in the USA is offering expertise and infrastructure to create and improve exposome evaluation tools.	(133, 134)
CANUE	The Canadian Urban Environmental (CANUE) Health Research Consortium understanding urban living and human health	(149)
SALURBAL	Salud Urbana en América Latina (SALURBAL), Urban Health in Latin America studying how urban environments and urban policies impact the health of city residents throughout Latin America.	(150)
**Exposomes databases and tools**		
T3DB	The Toxin—Toxin Target Database provides information about toxic exposomes including toxic materials that exist in air, water, or food.	(137, 138)
CTD	Comparative Toxicogenomic Database	(139)
EPA CompTox	Chemical dashboard	(140)
NORMAN Network	Provide information of compounds in the environment	(141)
NIOSHTIC-2	A bibliographic database	(142)
Exposome Explorer Database	Provide relationships with internal and external exposures	(143–145)
Blood Exposome	Provide relationships with internal and external exposures	(143–145)
ExposomeShiny	Emerging software toolbox using R code for implementing exposome data analysis.	(146)

## Understanding omics

The term “omics” refers to a set of techniques for studying the functions and interactions of organisms' molecular makeup (genomics, epigenomics, and transcriptomics) (85). More importantly, omics can provide research with a tool to provide surrogate measures for environmental exposures (biological monitoring of exposure), uncover the combined role of genetics and environmental exposures in human health outcomes and provide supporting molecular evidence to demonstrate the phenotypic effects of exposure and/or temporal pathology of disease or poor health outcomes (86, 87). Studies that provide a platform for researching genetic diversity, environment, lifestyle, and chronic disease will aid in the resolution of key aetiologic concerns during the next two to three decades (88, 89). These investigations, despite their high cost, are supported at least in part, by the belief that unraveling complex environmental and genetic etiologies will enable the adoption of effective public health treatments (90).

Omics technology needs to be adjusted in terms of sensitivity, sample size, throughput, and cost (91). Purification processes are critical in metabolomics and proteomics to allow investigation of less abundant but potentially more revealing proteins or metabolites in the context of more quantitatively dominant species (92). Just as with the last generation of exposure biomarkers, a well-organized approach, starting with model systems and small-scale human studies is likely to be the most successful (93).

### Genomics

With the advent of modern sequencing technologies, we are slowly drifting away from the gene-centric notion that genetic variants are the sole contributor to disease risk and clinical outcome (94). It has long been known that phenotypic variation of complex traits is derived from both genetic and environmental influences (95). In fact, this paradigm has been repeatedly represented as the equation G + E = P whereas G represents the genome and E represents its environmental counterpart (96). The study of how both genes and the environment interact not only allows us to elucidate the reasons behind different phenotypes but also gives us the chance to predict future phenotypic development. Genomic studies have helped identify how certain genomic variations expose different individuals to certain diseases (97, 98).

For instance, a meta-analysis of 2,748 twin studies discovered that the environmental influence on hundreds of complex human traits was almost as great as the genetic influence (99). Moreover, a research on monozygotic twins discovered that the average risk associated with 28 chronic illnesses was just 19% (94). Rapid advancement in genomics has been made possible by the highly structured character of the genetics discipline and by massive projects like the Human Genome Project that have sparked research and the creation of low-cost, high-throughput sequencing technology (100). Yet this understanding is curbed by the limits of heritability and transmission of genetic variants, in order to attain a complete picture, exposure tracking and integration of the exposome and the genome remains crucial. Novel integrative analysis methods are being developed to integrate genomic and exposomic data and attempt to identify the link between them (101).

### Epigenomics

Epigenetic modifications that affect the genome without altering the underlying DNA sequence impact gene expression (102). These alterations, which come about as a result of DNA methylation (or associated processes) or histone modifications, lead to long-term alterations in gene expression, which can endure during cell division and be passed down to succeeding generations (103). Various stressors, including chemical exposures, wounds, illnesses, and infections, can leave behind distinctive epigenetic markers that endure for a long time (104). Epigenomics is therefore an important method to assess exposure history and its impact on disease outcome (103). Different methylation patterns have been linked to chemical exposures in epigenome-wide association studies, shedding light on the processes driving biological responses and illness (105–107). Unfortunately, epigenomic studies have been so far characterized by studying relatively simple or single exposures. A more robust framework is highly needed to put in perspective/fully characterize the interaction between the exposome and epigenome.

### Metabolomics

The field of metabolomics, which entails the simultaneous and quantitative detection of large numbers of small molecules in biological systems, opens up new possibilities for understanding biological phenotypes, deciphering mechanisms, and identifying biomarkers or drug targets for a wide range of diseases (93, 108, 109). Traditionally, studies have focused on measuring the chemical space in human habitats using spectrometry tools such as mass spectrometry (MS) hyphenated with various chromatography approaches, such as gas chromatography (GC)-MS, liquid chromatography (LC)-MS or capillary electrophoresis (CE)-MS, or nuclear magnetic resonance spectroscopy (NMR) (110, 111). While NMR-based processes have a number of distinct benefits due to recently enhanced and high throughput instrumentation, MS's better sensitivity and larger chemical coverage are revolutionizing our capacity to study ambient metabolomes with higher throughput (92, 112, 113). Due to its non-selectivity, low cost, and ease of sample preparation, NMR spectroscopy-based metabolomics has clearly been the most popular analytical tool for environmental metabolomics studies during the last decade (112, 113).

### Proteomics

Proteomics is a powerful technique used to systematically uncover biological processes, metabolic networks, and functional protein interaction networks of cells and organisms under stress (114). Additionally, proteomics can be used to analyze the toxicity of pollutants and byproducts in order to early detect potential environmental risk factors and determine the success of pollutant treatment (115). The evolution of life has been influenced by environmental change to a vast extent (116). Proteins with a wide range of functions and structures have evolved over billions of years in response to environmental changes or stress (117). Nonetheless, our understanding of protein evolution has improved as genome data, protein sequence and structure and computing capacity have grown (117, 118). The intrinsic relationship between environmental succession, functional protein evolution, and proteome expression aids in the understanding of life's evolutionary processes (119).

The structural properties of proteins, as well as the physicochemical connections between effective proteins and pollutants, are thought to have a crucial role in the binding, absorption, and transformation of pollutants (120). The formation of adducts, alteration of phosphorylation status (Phorbol Esters), alteration of thiols, reactive oxygen species (ROS), and conversion of side chains to aldehyde or ketone groups are all examples of how environmental toxicants, medications, and diet interact with proteins (121). As a result, it is crucial to investigate the conformational changes and, as a result, biological effects of target proteins, and the effects climate change has on the environment (122). One strategy for uncovering the mechanism of protein evolution under stress is to compare the proportion of individual amino acid residues in the functional protein sequence at different stages of environmental stress and then calculate the correlation between the proportions of amino acid residues and protein physicochemical properties (123). Another technique for investigating the evolutionary process is to analyze the mutation site and conservation sequence of proteins at various phases of environmental stress (120).

### Transcriptomics

In the post-genomic era, transcriptomics became one of the most advanced fields. The transcriptome is the entire set of RNA transcripts in a certain cell type or tissue at a specific developmental stage and/or physiological condition, including messenger RNA, transfer RNA, ribosomal RNA, and other non-coding RNAs (124, 125). Transcriptomics focuses on gene expression at the RNA level and provides genome-wide data on gene structure and function to uncover the molecular mechanisms underlying certain biological processes (126, 127). In one study, Spira et al. evaluated gene expression in bronchial epithelial cells in smokers, ex-smokers, and non-smokers, which was of particular relevance because it sampled from the target cells (128). The groups differed in a variety of domains, including oxidant stress and glutathione metabolism, xenobiotic metabolism, and secretion (128).

### Other integrative approaches (the multi-omic approach)

Of note, other integrative approaches have started to take shape incorporating exposome studies with multiple omic data. For example, a recent study suggested the skin interactome model, a holistic combination of genomic, exposomic, and microbiome data (65). In this model, the authors computationally analyzed the relationship between various exposures, genetic factors and microbial flora and skin aging (65). While the model is still lacking strong experimental data to support it, it poses an interesting notion where the human microbiome should be integrated with other omics and exposure technologies.

## Recent exposome initiatives, projects, and databases

There have been exerted efforts and initiatives in terms of measuring all the exposures throughout an individual's life course (129). One of them is the EXPOsOMICS, which is an European project that mainly assesses environmental exposures particularly water and air pollution (130). It depends on finding relationships between external environmental exposures and biochemical and molecular alterations in the same person (130). Another European initiative is the HELIX project, which concentrates on the early-life exposome of mothers and children (131, 132). It gathers information about the environmental hazards that face mothers and children and reflect this knowledge to the health and development of younger generations (132).

On the contrary, the HEALS project aims to assess the internal exposure of individuals by incorporating the omics with biochemical biomonitoring data (133). The HERCULES project that was launched in the USA is offering expertise and infrastructure to create and improve exposome evaluation tools (133, 134). Although the field of exposome is very evolving and appealing, it is complicated. This is due to the missing information of environmental exposures indicators that are required for the omics model (135). Furthermore, to ensure the efficiencies of the exposomes approaches, systematic simulation, correlation studies, and statistics should be done (17, 136). There have also been several emerging exposome databases, toolbox software that enhances our understanding of total internal and external exposure assessment. The Toxin—Toxin Target Database (T3DB) is a database that provides information about toxic exposomes including toxic materials that exist in air, water, or food (137). T3DB is composed of toxic substances data associated with their toxic effects, chemical properties, mechanisms and their mode of actions, and gene expression datasets (138). The Comparative Toxicogenomic Database (CTD) and EPA CompTox chemical dashboard which comprises 882,000 chemical substances along with the NORMAN network provide information of compounds in the environment (139–141). On the other hand, NIOSHTIC-2 is a bibliographic database that offers occupational safety and health reports (142). It reflects the exposure-response and risk assessment field in the exposome and health (142).

Exposome Explorer and Blood Exposome are recent databases that provide relationships with internal and external exposures (143–145). Moreover, an emerging software toolbox such as ExposomeShiny has been designed using R code for implementing exposome data analysis (146). It supports researchers to observe patterns of interest in the data and integrate packages of new methodologies to cope with the field at high standard levels of quality (146). All of these tools and initiatives positively affect not only the health but also the financial mechanisms (147).

Globally, a plethora of projects are adopting exposomic approaches toward analyzing the impacts of environmental exposures on human health. For instance, in Canada, The Canadian Urban Environmental (CANUE) Health Research Consortium is working on understanding urban living and human health (148). CANUE's has partnered with Canada's cohort/health databases enabling unheard-of advances in our understanding of the interplay between environmental factors and health outcomes (149). This effort will make it possible to plan for healthy communities and towns in the present and the future using efficient, evidence-based techniques (149). A similar initiative started in Latin America under the name SALURBAL meaning Urban Health in Latin America, a 5-year project aimed at studying the interaction between urban environments and policies and their impact on human health (150). The project is supported by the Wellcome Trust and is initiated by The Drexel University Dornsife School of Public Health and partners throughout Latin America and in the United States (150).

Other nations are working on initiating dialogue concerning the exposome and the importance of utilizing exposure insights as a guideline for policies on human health. In Japan, The Gunma University Initiative for Advanced Research (GIAR, Japan) organized a workshop to debate the scope of exposomics from a global and interdisciplinary viewpoint (151). The results and insights from the workshop were published as a guideline to support other institutions who may wish to follow in their footsteps (152). A similar publication resulted from symposia held in Ottawa in 2018 by the ISEE (153). The publication summarized the outcomes of the Africa Chapter symposia which included Climate Change, Air Pollution and Environmental Health, and Exposure to Pesticides and Heavy Metals (153).

## The OMIC approach to monitoring climate change—Exposome interactions

The advancement of technology in nucleic acid sequencing, mass spectrometry and metabolic research has made it possible and faster than ever before to conduct omics research in biology. Integrating omics data from multiple biological levels of complexity (i.e., multi-omic incorporating genomics, transcriptomics, proteomics, and metabolomics) is now a feasible study design that has been adopted in a plethora of research topics (154). Furthermore, recent technological advances in monitoring natural, social, and personal environments have opened the door to a more comprehensive assessment of the exposome than has previously been possible (155). Handheld and wearable devices, which are capable of assessing geographic location, personal movement, real-time questionnaire responses, and other aspects of the personal environment, are likely to have the greatest impact on the assessment of the personal environment (156). As new sensors are developed to measure other indicators in real time and are integrated into devices such as smart watches, they may be able to provide a more comprehensive evaluation of the individual's immediate personal exposures in future studies (157).

Climate change monitoring has also undergone massive breakthroughs of its own with the availability of satellite remote sensing of essential climate variables (ECV) (158). Linking ECVs with changes in exposures has also become easier. Consider the NASA TEMPO satellite, which is scheduled to launch in 2022 and will be able to assess a variety of hazardous air pollutants on an hourly basis with significant spatial variability (159). The use of unmanned air vehicles (UAVs) will also make it easier to monitor emissions and quality of air, adding yet another layer of detection in the integrated monitoring of climate-change-induced exposure variations (160).

**Figure 2:**
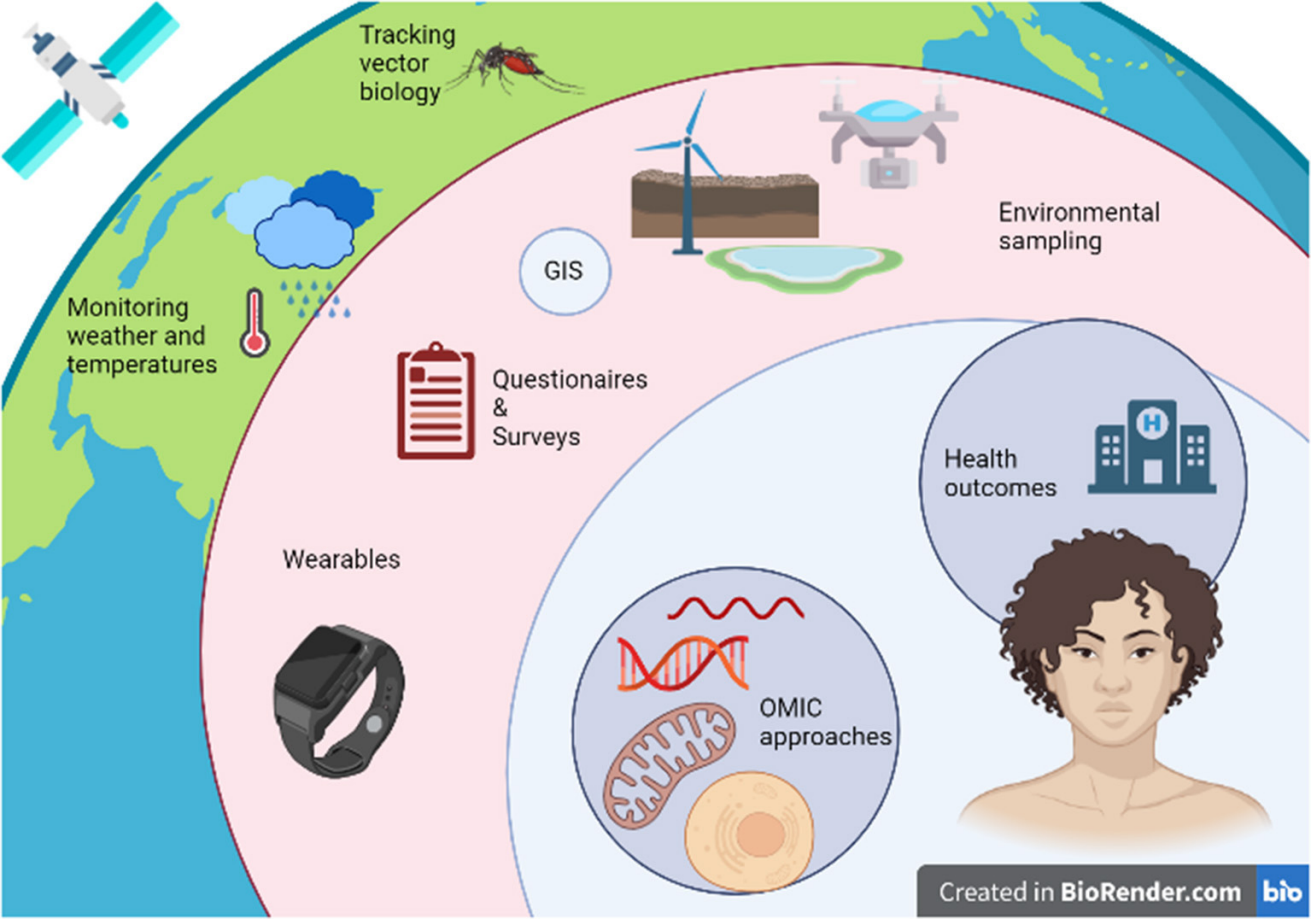
Integrating omic, exposome, and climate change research to assess the impact of climate change-induced environmental exposures on human health.

While these advancements pave the way to the creation and implementation of innovative study designs to evaluate the impact of climate change-induced exposures on human health as in Figure 2, several challenges will arise particularly in the area of data analysis. The large volumes of data derived from newly developed technologies, will require novel tools to characterize and analyze them in efficient ways. Researchers will need to step back from simplistic statistical methods and develop new tools to consider the results of said studies in a holistic manner (161). Advances in artificial intelligence and machine learning may provide a basis for understanding, linking, and processing complex systems that are beyond the capabilities of humans, with the ultimate goal of developing and implementing effective public health policies (162). When these new forms of analysis are applied to an exposome approach to disease, they have the potential to yield novel information about the climate-change-human-environment interface as well as new insights into the etiology of numerous diseases (163).

### Understanding the technical challenges

A significant impediment that is hindering the design and implementation of large-scale exposomic/multi-omic studies is the methodological challenge. For instance, there exists a wealth of spatial and contextual exposure data that is unfortunately highly complex in comparison to individually-measured exposomic factors (164). Such challenges include the difficulty of exposomic data engineering and spatiotemporal data linkage as well as the difficulty of selection of appropriate statistical methods and the need for advanced machine and deep-learning methods for data analysis (164–166). For instance, a recent study investigating the relationship between early-life exposures and childhood obesity utilizing an exposome-wide approach struggled with many hindrances including the cross-sectional nature of parts of their data, the heterogenous nature of their data that was acquired using different types of measurement errors, the lack of technology supporting them in acquiring a true and full exposome and finally residual confounding and misrepresentation of the population could not be excluded (167).

Methods to assess external exposures such as geographic information systems; remote sensing; global positioning system and geolocation technologies; portable and personal sensing, and self-reported questionnaire assessments are all available. A study examining the external exposome and COVID-19 mortality was recently published and showed that exposure variables including air pollutants, vacant land measures and food environment measures showed significant associations with COVID-19 mortality (168). While these methods of tracking external exposure have strong potential of providing valuable insights, they come with some shortcomings and priority areas of research for their enhancements as well as recommendations have been outlined elsewhere (169).

### Asking the right questions

It is evident that in order to utilize the growing arsenal of technological tools made available to today's researchers to address the interaction between climate change-induced environmental exposures and human health, one must first begin to ask the appropriate questions. Such questions must shape the experimental designs of the near future if we are to keep up with the alarming and rapidly progressing nature of climate change. Furthermore, these questions must aptly utilize the omic tools that are available at our disposal as well as attempt to overcome the shortcomings of previous evidence. A suggestion for such questions is as follows:

1. How are different CCIEEs related to human disease risk, prevalence, progression, and events? Can we outline this difference using multi-omic approaches incorporating modern exposome tracking and analysis techniques identified in this review?
2. Which specific domains of interaction between humans and CCIEEs carry the most weight in terms of impact on human health in the context of the exposome and the multi-omic paradigm?
3. What are the potential interactions themselves CCIEEs and do these interactions exacerbate their risks or mitigate them? If these interactions do exist, can we track them on multiple omic landscapes?

To answer these questions, not only do highly complex multi-omic study designs need to be implemented accompanied by innovative data analysis tools but also the quality and rigor of the data must be ascertained. Data must be measured at accurate intervals and most importantly, longitudinal life-long studies are direly needed to estimate the impact of exposure to CCIEEs. Furthermore, it would be highly beneficial to map out the interactions between different CCIEEs through a network analysis similarly to the environmental interaction maps generated in ecosystem modeling (170).

### Policy and the multi-omics approach

While the world hopes to create strategies to mitigate climate change, strategies for adaptation are direly needed to predict and cope with the results of this phenomenon. Either type of strategy must to be guided by insights into the potential effects of climate change-induced environmental exposures on human health (171). As such, the integration of technological advancements in the fields of climate/environmental monitoring, exposome studies (to monitor changes in exposure), and OMIC analyses (to monitor molecular changes associated to varying exposures) is recommended (171). This integration should be within the framework of well-structured longitudinal study designs that monitor each of these layers of complexity. It will require inputs from all sectors of government and civil society to manage the health effects of climate change (172). It will also require collaborative efforts between many academic disciplines, as well as innovative international cooperation models (172). A critical component of the adaptation process will be the monitoring, mapping and prioritization of which health impacts to tackle first during the adaptation process. An integrated and multidisciplinary approach to reducing the negative health effects of climate change necessitates the implementation of at least three levels of intervention (79). First and foremost, policies must be implemented to reduce carbon emissions while simultaneously increasing carbon bio-sequestration, thereby slowing global warming and ultimately stabilizing temperatures. Second, action should be taken in response to the occurrences that have been linked to climate change and disease (79). Third, appropriate public health systems should be put in place to deal with the consequences of poor health outcomes (172).

## Conclusion and future perspectives

With the current anthropogenic shifts that are affecting our planet's health, science is attempting to promptly identify, assess and mitigate risks to the health of our planet and their potential impacts on human health. Climate change is an alarming phenomenon that threatens to change life as we know it. There is no doubt toward the impending health repercussions associated with changes in exposure due to climate change.

With the advent of innovative technologies, scientists have been able to explore never-before-seen landscapes within human bodies and cells. Whole disciplines emerged dedicated to the study of the whole, omics examine the cell's entire genome (genomics), whole RNA content at a given moment in time (transcriptomics), the entirety of non-genomic modifications (epigenomics), entire protein content (proteomics), and even the full cellular metabolic profile (metabolomics). Yet the culmination of these technologies pushed researchers to consider these cellular characteristics within the context of the environment leading to the concept of the exposome, the full history of human environmental exposures.

Now more than ever, novel study designs that incorporate multiple types of said analyses are needed. Integrating multiple omics approaches with exposomic tracking will generate a wealth of precious data. The development of novel and innovative methods to analyze the resulting data utilizing advancements in the fields of artificial intelligence and machine learning will allow the optimization of the utilization of this data. Results of this type of studies would provide invaluable insights into the health effects of climate change and its associated phenomena. This would help guide novel policies and guidelines for the mitigation of these risks and effects and allow us to navigate an era of uncertainty.

## Author contributions

All authors contributed to the conception and design of the study, wrote the first draft of the manuscript, and wrote sections of the manuscript. HA, WA, and AA organized and provided feedback on the writing of the manuscript. All authors contributed to manuscript revision, read, and approved the submitted version.

## Conflict of interest

The authors declare that the research was conducted in the absence of any commercial or financial relationships that could be construed as a potential conflict of interest.

## Publisher's note

All claims expressed in this article are solely those of the authors and do not necessarily represent those of their affiliated organizations, or those of the publisher, the editors and the reviewers. Any product that may be evaluated in this article, or claim that may be made by its manufacturer, is not guaranteed or endorsed by the publisher.
